# A Systematic Review of E-Waste Generation and Environmental Management of Asia Pacific Countries

**DOI:** 10.3390/ijerph18179051

**Published:** 2021-08-27

**Authors:** Lynda Andeobu, Santoso Wibowo, Srimannarayana Grandhi

**Affiliations:** School of Engineering and Technology, Central Queensland University, 120 Spencer Street, Melbourne 3000, Australia; s.wibowo1@cqu.edu.au (S.W.); s.grandhi@cqu.edu.au (S.G.)

**Keywords:** e-waste, environmental management, challenges, health impacts, Asia Pacific

## Abstract

Due to the rapid increase in the use of electrical and electronic equipment (EEE) worldwide, e-waste has become a critical environmental issue for many governments around the world. Several studies have pointed out that failure to adopt appropriate recycling practices for e-waste may cause environmental disasters and health concerns to humans due to the presence of hazardous materials. This warrants the need for a review of the existing processes of e-waste management. In view of the growing e-waste generation in the Asia Pacific region and the importance of e-waste management, this study critically reviews previous research on e-waste generation and management practices of major e-waste producing nations (Australia, China, India, Indonesia, and Malaysia) in the Asia Pacific region, provides an overview of progress made and identifies areas for improvement. To fulfil the aims of this research, previous studies from 2005 to 2020 are collected from various databases. Accordingly, this study focuses on e-waste generation and environmental management of these countries. This study found that e-waste management practices of the selected countries need to be enhanced and recommends several best practices for effectively managing e-waste.

## 1. Introduction

The Asia Pacific region is highly populated and is considered one of the fastest developing regions in the world. In addition, many countries in this region underwent rapid industrialisation, driven by foreign direct investments [1] due to a relatively cheap labour force. One of the industries that benefited from these factors is the electrical and electronics industry, which has experienced a major transformation due to increased technological and market developments [2]. Today, electrical and electronic equipment (EEE) has become indispensable and enhance living standards, but often contain toxic chemicals that negatively impact human health and the environment and fuel the climate crisis [2,3]. The growth in demand and increased sales of EEE have consequently led to the rise in the volume of e-waste [3,4,5].

E-waste is one of the most urgent and pressing challenges of our time; however, it is routinely ignored. Across the world, the growing amount of e-waste threatens the environment and local communities, as incorrectly disposed e-waste results in life-endangering toxic chemicals released into the environment and the loss of precious metals [2,4,5,6,7]. Perkins et al. [8] point out that the amount of e-waste generated each year is increasing at an alarming rate. In 2019 alone, more than 50 million tons (Mt) of e-waste was generated globally. Of this total e-waste, 24.9 million tons were generated in the Asia Pacific region alone. The amount of e-waste generated worldwide increased three times faster than the world’s population. Forti et al. [2] estimate that the volume of e-waste generated globally will exceed 74 million tons (Mt) by 2030. However, the level of recycling is not keeping up the pace. In fact, less than 13 per cent of e-waste was recycled in the same year. Moreover, the majority of e-waste generated is being diverted for landfilling, which is a common approach to disposing of e-waste worldwide [9]. The major issue with the current e-waste management practices is: (a) lack of efficient collection and recycling systems and (b) lack of mechanisms to hold producers of EEE accountable for the end-of-life disposal [2]. Hence, failure to adopt appropriate e-waste recycling processes may lead to enormous environmental and health issues [3,10,11,12,13].

This study identified three research gaps. Firstly, although, literature presents results of various studies on e-waste generation [3,4,5,8,14,15,16,17], recycling [14,15,16,17], treatment [4,18,19,20], and environmental management [8,21,22,23,24]; however, few studies have focused on the impact of e-waste generated in the Asia Pacific countries selected and its consequential effects on human health and the environment. Secondly, Forti et al. [2] suggest that many countries, including countries in the Asia Pacific region, are not sufficiently managing e-waste generated, and greater effort is needed to ensure smarter and more sustainable global production, consumption, management, and disposal of e-waste. The authors also indicated that more e-waste is generated than is being safely recycled in many countries of the world, and more corporative efforts are needed to tackle the escalating e-waste problem through appropriate research and training. Forti et al. [2] and Balde et al. [3] noted that the issues emanating from e-waste management in today’s digitally connected world are primarily due to the way we produce, use, and dispose of electronic devices, which are currently unsustainable. Bhaskar and Kumar [25] added that implementing appropriate e-waste management strategies will contribute to the achievement of sustainable development goals and reduce the global climate crisis through developing the necessary, needed, and required e-waste policies. Thirdly, while investigations and discussions on e-waste generation and management have been ongoing for several decades. However, the problems and challenges on e-waste generation and management remain unabated [2,26,27].

The purpose of this study is to critically review the existing strategies and practices adopted by the major e-waste producing countries in the Asia Pacific region in managing and regulating e-waste to minimise the environmental and health impacts created as a result of inappropriate recycling and disposal practices.

A key initiative and motivation of this study is to identify the problems/challenges in managing e-waste in the selected Asia Pacific countries and recommend appropriate management strategies and policy approaches to handle and regulate e-waste to significantly reduce environmental and health concerns. Accordingly, this study reviews previous research on e-waste generation and environmental management of Australia, China, India, Indonesia, and Malaysia, identifies problems and challenges that negatively impact e-waste management in these countries, provides an overview of progress made, and identifies areas for future research.

The selected countries (Australia, China, India, Indonesia, and Malaysia) are among the largest producers of e-waste in the Asia Pacific region [2,13,18,28]. To fulfil the aims of this study, a comprehensive review of previous research articles on e-waste published from 2005 to 2020 was conducted. This study focuses on aspects such as the amount of e-waste generated, current recycling and disposal methods, environmental management of e-waste, individual/collective attitudes towards e-waste, current e-waste problems/challenges of selected countries. In addition, prior studies of the selected countries are categorised based on the type and scope of research, location of study, and e-waste categories analysed. This study uses the outcomes of previous studies, considers country-specific issues, and identifies future research areas to present best practices for e-waste generation and environmental management.

This paper is organised into five sections. The first section presents current literature on e-waste, the research problem, research gaps and research aim, and justification for this study. The second section outlines the chosen methodology and the justification for considering a systematic literature review. The third section details the e-waste management practices in the selected countries. The fourth section provides the results of this study and analyzes the results. The final section presents the findings of this study, limitations associated with the current study, policy recommendations for effective e-waste management, and future research opportunities.

## 2. Research Methods

In recent years, researchers have increasingly used quantitative and qualitative research (mixed methods) techniques to expand the scope and improve the analytic power of their studies [29,30]. Quantitative research method is a statistical and interpretive technique used to describe or explain the meaning and relationships of a phenomenon under investigation. Quantitative research typically involves probability sampling to allow statistical inferences to be made [29,31]. In contrast, qualitative research method is a non-numerical, precise count of some behaviour, attitudes, knowledge, or opinion for ascertaining and understanding the meaning and relationships of certain phenomena for generalisation. It typically involves purposeful sampling to improve understanding of the issues being examined [29,30,31].

This study adopts a qualitative research method to explore the issues relating to e-waste in the selected countries from existing research over the past years to guide future research in this area. To achieve the aim of this study, the five-phase approach of Wolfswinkel et al. [32] for conducting a systematic review and analysis of the literature is adopted. Adopting this five-phase approach enables the researchers to conduct a thorough search process and critically review and analyse the articles retrieved from the databases. The five-phase approach includes: (a) defining the scope of the review, (b) searching the literature, (c) selecting the final samples, (d) analysing the samples using content analysis, and (e) presenting the findings.

The first phase is to define the scope of the review. This includes the definition of specific criteria for the inclusion and exclusion of relevant sources and the criteria for identifying and retrieving those sources in the literature. In this study, four prominent databases are used to source literature, including ProQuest, Emerald, ScienceDirect, and Web of Science. The selection of these databases is due to their representativeness and coverage in the publication of top academic papers on e-waste in the selected countries. To ensure broad coverage of the studies in these databases, several keywords have been used for the search, which includes “electronic waste”, “e-waste”, “waste electrical and electronic equipment”, “e-waste management”, “e-waste recycling,” “e-waste disposal methods”, “e-waste problems and challenges” and “environmental management of e-waste”. Several criteria are used to set the limitation, including restricting the document type to scholarly journals, peer-reviewed conference papers, book chapters, and other institutional reports from United Nations (UN) and World Health Organization (WHO); the language in English, and the publication date from 2005 to 2020. These document types have been selected as they represent state-of-the-art research outputs with high impact [32].

The second phase is to run the search query within the selected databases for retrieving the search results. A total of 688 articles are returned using the above pre-defined search strings. This initial search enables us to gain a general understanding of the coverage of e-waste topics.

The third phase involves selecting the final samples for detailed analysis. The search is limited to the title and the abstract to focus on the search results. Titles and abstracts of all initial articles are screened for checking the relevance to e-waste. This leads to the identification of 235 relevant articles. Duplicate articles are removed. A total of 210 articles is assessed for eligibility, and after excluding those articles that did not meet eligibility criteria, a total of 185 articles is identified for further review.

**Figure 1:**
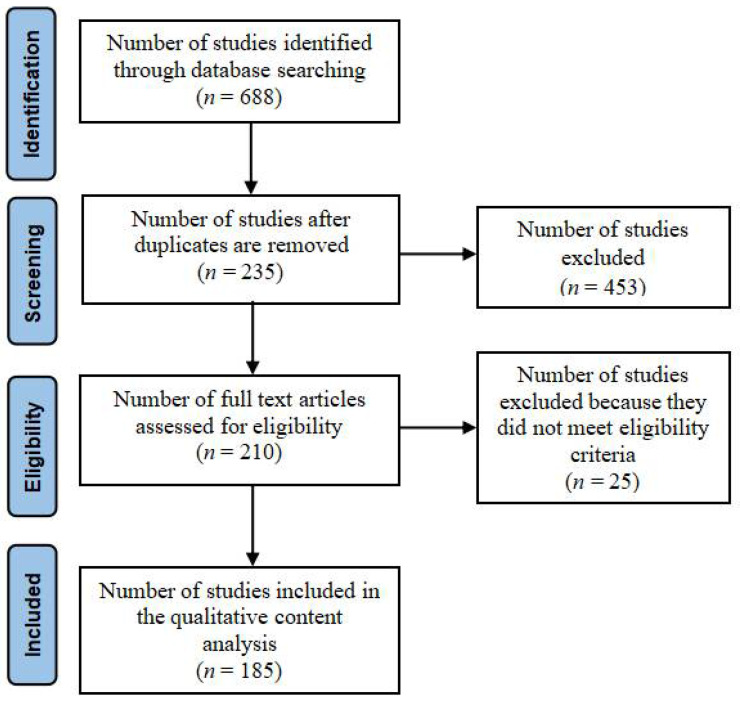
PRISMA flow chart indicating the results of searches.

The 185 articles have been read in full for coding and analysis. NVivo 12.0 is used for providing an overview of the general topics from all the abstracts of the included papers. An overview of the dispersion of the selected papers in terms of year of publication shows there is increased interest in e-waste from 2005 to 2020. Figure 1 below illustrates the search process using the PRISMA flow diagram.

## 3. Overview of E-Waste

E-waste is defined as an electrical appliance that no longer satisfies the user for its intended purpose [33]. Meanwhile, StEP [34] defines e-waste as a term used to cover items of all types of EEE and its parts that have been discarded by the owner as waste without the intention of reuse.

Table 1 shows e-waste generated around the world and per continent in 2016. It is observed that the Asian continent generated the highest e-waste, followed by Europe and the Americas. Interestingly, the African continent produced one of the lowest e-waste even though it is the second most populated continent in the world [35]. Although the African continent produced the lowest amounts of e-waste due to slow technological growth and limited access to energy when compared to other continents, they suffer other kinds of pollution problems caused by traffic emissions, oil spills, heavy metals, refuse dumps, dust, and open burnings and incineration, which significantly contribute to environmental contamination in Africa [36,37,38]. Human exposure to toxic metals and environmental pollution has become a major health risk in Africa and is the subject of increasing attention to national and international researchers and environmentalists [37,38].

A further study was conducted in 2019 whereby the Asia Pacific region also generated the highest amount of e-waste in comparison to America, Europe, Africa, and Oceania regions. The Asia Pacific region generated around 25 Mt, followed by America at 13.1 Mt and Europe at 12.1 Mt. The study also showed that Africa generated 2.9 Mt and Oceania generated 0.7 Mt of e-waste [2,39]. This warrants the need to conduct a study on e-waste generation and environmental management of countries in the Asia Pacific region [14,15,40].

### 3.1. Constituents of E-Waste

Over the years, the use of electronic devices for domestic and commercial purposes has grown rapidly [8]. E-waste generally consists of a range of hazardous materials (Table 2), including metals, pollutants, printed circuit boards, computer monitors, cables, plastics, and metal-plastic mixtures [2]. The composition and quantities of these materials vary in each electronic device depending on the manufacturer, the equipment type, model, and the age it was discarded. In comparison to household e-waste, the e-waste from the IT and telecommunication sector generally contains metals that are of high economic value [41,42]. These metals are generally categorised into precious and toxic metals. Precious metals include gold, silver, aluminium, iron, copper, platinum, etc. The value of precious metals in e-waste is estimated to be worth USD 14 billion. However, more than 50 per cent of these metals are not recovered [2]. Meanwhile, toxic metals in e-waste include mercury, cadmium, lead, and chromium [2,43].

### 3.2. E-Waste Generation and Management Practices

This study has selected five countries, including Australia, China, India, Indonesia, and Malaysia, from the Asia Pacific region because they are the major e-waste producers in the region. In line with the aim of this study, this section presents an in-depth analysis of waste generation, policies and management practices adopted by the selected countries in the Asia Pacific region. In addition, this section presents literature on e-waste generation and the opinions of scholars in this field. The following sub-sections explain e-waste management practices for the selected countries in the Asia Pacific region. Table 3 below presents e-waste key statistics for the selected countries.

#### 3.2.1. Australia

Australia is placed among the top 10 consumers of electronic products in the world. As a result, e-waste has become one of the fastest-growing waste streams in Australia [9,44,45]. The total and per capita e-waste generation in Australia has steadily increased in the last 10 years from 410 Kilotons (Kt) in 2010 to 554 Kt in 2019 as a result of an increase in sales of EEE [2]. Previously, due to the lack of an e-waste national regulatory framework, local government councils had difficulties in managing e-waste, and they had no strategies to address e-waste issues [46,47]. To resolve the nation’s escalating e-waste challenges, the Australian government established the National Waste Policy in 2019 to integrate existing policies and regulatory frameworks for e-waste management [9,45,48]. Thereafter, the Australian government introduced the National Product Stewardship Scheme in 2011 in collaboration with the State and Territory Governments and industries [9,26,45].

The introduction of the National Waste Policy in 2009 was designed to set the direction of Australia’s e-waste management and resource recovery for 10 years from 2010 to 2020. The policy was established to achieve several goals, including compliance to international obligations such as the Basel and Stockholm Conventions, reducing the generation of e-waste, and ensuring e-waste treatment, disposal, recovery, and reuse is safe and environmentally sound [44,47]. The Product Stewardship Act of 2011 was also designed to establish a framework by which the environmental, health, and safety impacts of electrical and electronic equipment and its recycling and disposal are adequately managed [44,45]. Currently, Australia’s e-waste system is in its evolving stages and while, progress has been made since the introduction of the National Waste Policy and the Product Stewardship Act, Australia’s e-waste is growing three times faster than other waste streams, and the capacity and sophistication of the nation’s systems need to grow and adapt [44,48].

#### 3.2.2. China

China is one of the leading producers of EEE, and currently, the country is experiencing incredible growth in e-waste generation from both domestic and international sources [9,26,49]. Formal e-waste management in China is driven by government agencies designed to improve e-waste recycling and disposal and to encourage manufacturers to take back their products [21,49]. Thus, Chinese e-waste regulations are focused on extended producer responsibility (EPR), polluter pays, and 3Rs (reduce, reuse, recycle) principles [50].

Informal e-waste recycling in China is often carried out by individual recyclers and unauthorised dismantling companies. Informal recyclers purchase used items and often either dismantle or repair them for the second-hand market. This unregulated e-waste recycling method is currently flourishing in China. Informal recycling provides livelihoods for many Chinese citizens and is creating serious environmental and health concerns. Thus, e-waste generation and management in China has remained a major problem and are fuelled by China’s inexpensive labour and manufacturing abilities. Informal recyclers do the majority of e-waste collection and recycling in most cities throughout China [50].

#### 3.2.3. India

The increasing average annual growth rate from 0.56% in 1991 to 1.62% in 2011 has contributed significantly to an alarming amount of e-waste generation in India. India is among the top 10 countries in the world in e-waste generation after the U.S. and China. It is estimated that three (3) million tons of e-waste were produced in 2018 and is expected to reach five (5) million tons by the end of 2020 [51,52,53]. According to the Confederation of Indian Industries, the Indian electronics industry has a market size of approximately USD 65 billion in 2013, and this is expected to reach USD 400 billion by the end of 2020 [52,54].

Today, e-waste in India is a significant waste stream both in terms of volume and toxicity [55]. Approximately 152 million units of computers will become obsolete in India by the end of 2021 [55,56], creating serious management challenges and environmental/health problems. Each year, India domestically produces approximately 400,000 tons of e-waste [24]. Thus, India’s e-waste recycling is a market-driven industry [55] and is dominated by a number of informal actors. About 90% of the e-waste in India is illegally recycled in the informal sector and involves different groups, including women and children [57,58].

The Ministry of Environment and Forests (MoEF) is the national regulator responsible for formulating legislation related to e-waste management and environmental protection. MoEF approves the guidelines for the identification of the various sources of e-waste in India and endorses the procedures for handling e-waste in an appropriate and environmentally friendly manner [59]. Those involving e-waste are the 2004 “Municipal Solid Waste Management Rules” and the 2008 “Hazardous and Waste Management Rules.” New regulations are classified as the 2010 “E-waste Management and Handling Rules”, which became effective in 2012 [60]. While there are regulations on e-waste management and disposal in India, no regulation has effectively addressed the e-waste problem in India [52,58]. Currently, the majority of the hazardous materials found in e-waste are covered under “The Hazardous and Waste Management Rules, 2011 and the 2016 E-waste Management and Handling Rules” [52].

Despite EPR being a major policy approach in both e-waste (Management and Handling) Rules 2011 and E-waste (Management and Handling) Rules 2016, they are not effectively implemented, and this can be attributed to certain peculiarities in India’s e-waste management system [51,61]. For example, due to some financial incentives involved, Indian consumers are willing to sell their obsolete e-waste to the “kawariwalas” (door-to-door scrap collectors). This behaviour is totally different from practices adopted by most developed countries whereby the producers and consumers have to pay “Recycling/Disposal Fee” [62,63,64].

#### 3.2.4. Indonesia

Due to substantial growth in the economy coupled with rapid technological developments, e-waste generation in Indonesia has increased considerably [28,65]. In 2016, Indonesia generated 1274 kt of e-waste with a per capita generation of 4.9 kg [66]. Although e-waste appears as a global issue, it is not a common term for most people in Indonesia [67,68]. In Indonesia, e-waste management is dominated by the informal recycling sector, which is essentially made of unregulated and unregistered small businesses, groups, and individuals, while the formal sector consists of the country’s municipal agencies as the major actors [69].

Although the country has no presence of a specific regulation to manage its e-waste, the “Environmental Protection and Management Act No. 32/2009” and “Solid Waste Management Act No. 18/1999” are used in the regulation of e-waste produced in the country [70,71]. Since 2016, the Indonesian government has been in the process of formulating a unified e-waste regulation for the country, which would apply to all the 37 Indonesian provinces, but this is yet to be realised [28,72]. However, the absence of regulated licensed recycling companies in the country has encouraged inappropriate disposal of the majority of the EEE from households, businesses, and industries [71]. Currently, the informal sector illegally collects, treats, and disposes of discarded EEE triggering huge environmental and health concerns [65,72]. The difficulties/challenges in managing e-waste in Indonesia is primarily due to (a) the inability of the government to understand and deal with the interest of stakeholders involved, (b) the government regulations are beneficial to only a few parties, and (c) there is strong resistance between the government agencies [73].

#### 3.2.5. Malaysia

In 2019, the International Monetary Fund (IMF), in its economic outlook, ranked Malaysia as the 3rd largest economy in Southeast Asia and the 37th largest economy in the world [74]. With a healthy economic indicator, e-waste generation in Malaysia is expected to increase in the coming years. The growth in e-waste generation is anticipated worldwide because there is a strong correlation between economic growth and e-waste generation [75,76].

Management of e-waste in Malaysia is still in its infancy and only began in 2005 [77]. In Malaysia, e-waste is classified as scheduled waste under the code SW 110, “Environmental Quality Regulations 2005” and managed by the Department of Environment (DOE) and the Ministry of Natural Resources and Environment (MNRE) [78,79]. The primary role of DOE and MNRE is pollution prevention and control through the enforcement of the “Environmental Quality Act 1974” (EQA 1974) [79,80]. Although there are strategies on e-waste management in place, they do not adequately guide the local consumers or the municipal authorities on how e-waste should be managed, reused, recycled, or disposed of [78]. Subsequent to the listing as e-waste under the “Environmental Quality Scheduled Waste Regulations (EQSWR) 2005”, e-waste in Malaysia was reported and managed as municipal solid waste through the Department of Solid Waste Management (DSWM) under the Ministry of Housing and Local Government [78,81,82].

### 3.3. A Review of Previous Studies

This study considered literature reviews to identify key issues associated with e-waste management and to conduct an extensive evaluation of e-waste management practices in the selected countries. We believe this knowledge will help the countries to overcome their challenges and develop appropriate strategies for recycling and disposing of e-waste. This section provides an overview of earlier studies in the selected countries. In particular, results from the literature review on e-waste generation and management practices adopted by the respective nations are presented. Furthermore, this section presents the scope and the context of earlier studies on e-waste management. Prior studies [83,84,85,86] offer valuable insights into e-waste management in the selected countries. They also highlight the challenges associated with e-waste management and the need for developing comprehensive e-waste management strategies. Table 4 presents previous research on e-waste conducted in the selected countries from 2005 to 2020.

## 4. Results and Discussion

This study adopts a qualitative approach for studying e-waste management practices of the selected countries in the Asia Pacific region. As per Wolfswinkel et al. [32], this study adopted a five-phase approach. In the first phase, secondary data from 2005 to 2020 has been considered for reviewing existing literature on e-waste management in the selected countries. Then, a total of eight (8) keywords are used to identify and analyse the relevant articles. Finally, challenges and practices associated with e-waste management are discussed to present the proposed policy approaches and recommendations.

E-waste management has become a contentious issue due to the presence of hazardous materials and the health hazards it may cause if not managed properly. In fact, for more than a decade, scholars have conducted studies on informal e-waste collection and disposal methods [87,88]. However, these studies were limited to e-waste generation, prevention, quantification, recycling, treatment, reuse, pollution control, legislation, and life-cycle assessment, as noted in recent studies [83,85,87,89,90,91]. Undoubtedly, these studies presented opportunities to address some of the challenges associated with e-waste management. However, there is a limited study in addressing the environmental and health implications associated with e-waste for achieving sustainable e-waste management. Moreover, prior studies on e-waste are centred on a small number of developed countries, which represent a “standard” or “benchmark” for developing e-waste management policies for emerging countries. Therefore, this study aims to address these gaps.

### 4.1. E-Waste Studies in Selected Countries

After a critical review of the pertinent literature and a content analysis of the e-waste articles related to the selected countries, the dispersion of e-waste research in the selected countries according to the keywords/themes, e-waste categories examined, and the study location are illustrated in Table 5. Based on the information presented in Table 5, it is evident that most of the e-waste studies in the selected countries were focused on e-waste generation, management and recycling. A number of e-waste studies focused on problems and challenges, environmental management, and health impacts indicating that further research is required in these areas in the countries examined.

### 4.2. Analysis of Content Results

Given the background review and analysis in the previous sections, it is obvious that the problem and challenges of e-waste in the selected countries still persist. Our analysis shows that the e-waste management systems and infrastructure of the selected countries, particularly India, China, Malaysia, and Indonesia, are still in their infancy. Currently, e-waste scrap such as printed circuit boards, CRT monitors, and LCD screens have been, and are still being, recycled in China, India, Indonesia, and Malaysia, creating huge environmental and health issues. Informal e-waste collection, recycling, and its health implications on informal workers in these countries have become increasingly popular in the last 15 years [89,92,93,94]. Table 6 shows the findings from the analysis of the contents.

In China, several towns have remained as a dumping ground for e-waste. For example, Guiyu town is often referred to as “the e-waste capital of the world” and employs more than 150,000 locals from four villages. These local informal workers dismantle and recapture valuable metals and parts that can be reused or sold from old computers. In Guiyu, it is not uncommon to see computer parts, cables, and huge tangles of wires scattered around the streets and riverbanks [88,95,96,97]. Findings/outcomes indicate that various issues geared towards developing a sustainable recycling system still need to be addressed.

In India, obsolete computers from households and businesses are sold by auction to door-to-door collectors who engage in informal methods of recycling. According to a report by the Confederation of Indian Industries (CII), approximately 146,000 tons of obsolete EEE are generated in India annually [86,109]. The results of the analysis show that the recycling of e-waste in India is heavily dominated by the informal sector, and only a few approved e-waste recycling facilities are available. In the majority of urban slums of India, more than 95% of e-waste is treated and processed by untrained workers who carry out illegal and risky procedures. These illegal procedures are not only injurious to the health of the locals who work without personal protective equipment but also to the environment [55,86]. It is found that the formal process of e-waste recycling and treatment is still rather slow as the collection and recycling of most e-waste remains in the hands of the informal sector [86,109].

In Indonesia, large amounts of e-waste are imported from developed countries. E-waste in the form of scrap materials or second-hand devices is sent to Indonesian islands from the adjacent ports in Singapore and Malaysia. Findings indicate that, in Indonesia, infrastructure and workable systems to quantify, recycle, monitor, and handle e-waste is lacking [65,127]. Currently, the informal sector illegally collects, treats, and disposes of discarded EEE, causing huge environmental and health issues [65,71].

The management of e-waste in Malaysia is still developing and only began in 2005 [77]. Results indicate that although there are strategies to manage e-waste in Malaysia, challenges persist and the pressure to manage e-waste is now even more crucial. Malaysia has become one of the popular destinations of e-waste imported from developed countries [139,140,141]. Results of the analysis also indicate the country still faces significant issues in managing the ever-increasing amount of e-waste generated even though several material recovery facilities (MFR) have been established.

In Australia, several government policies have been developed. The key issues are identified in the e-waste management including: (a) the narrow scope of e-waste categories for recycling, (b) the lack of clarity on the roles of key stakeholders involved, (c) the recycling and material recovery targets, and (d) the lack of auditing and compliance. The results of the analysis show [47,142,143] minimal research has been undertaken to assess the effectiveness of e-waste policy management strategies [47,144,145,146,147].

It can be seen that the majority of the selected countries in this present study are faced with an increasing amount of e-waste. Although the per capita e-waste generated in the emerging countries is much lesser than in the developing countries, the volume generated is greater due to the growing population and market size in emerging countries such as India, China, and Indonesia. These countries are ranked among the top e-waste generators in the world.

The importance of selecting these countries such as Australia, India, China, Indonesia, and Malaysia in the Asia Pacific region in terms of environmental and market perspectives cannot be overemphasised. These selected countries have significant population, natural resources, and financial potentials [67,148,149,150,151]. Moreover, these countries have contributed substantially to the world’s GDP, landmass, and market share. This calls for a responsible e-waste management effort by these countries to effectively manage the growing amounts of e-waste generated for reducing environmental and health concerns.

Clearly, e-waste management processes in the majority of these countries examined still need improvement. Most of these countries studied have no well-established e-waste infrastructure for efficient collection, storage, transportation, recycling, and disposal of e-waste. In addition, the enforcement of codes of practice and regulations relating to hazardous e-waste management in these countries is minimal or non-existent.

Exposure to e-waste is harmful to public health. E-waste has been found to negatively impact public health because communities are exposed to a complex mixture of chemicals from multiple sources and through multiple exposure routes [152]. The results of this study indicate that the impact of e-waste is linked to a variety of health problems in the countries examined, such as birth defects, premature births, respiratory diseases, and cancer. Furthermore, people living in e-waste recycling towns or working in e-waste recycling sites showed evidence of greater DNA damage. A review of the literature also revealed an association between e-waste exposure and thyroid dysfunction, adverse behavioural changes, and damage to the lungs, heart, and spleen due to prolonged exposure [152,153].

Hence, e-waste has become one of the major challenges in these countries, and it is, therefore, crucial for these countries to investigate the development of a well-organised and inexpensive recycling scheme to extract valuable resources with inconsequential environmental impacts.

## 5. Conclusions

This study has evaluated the e-waste generation and management practices of the selected countries in the Asia Pacific region. Based on the review of past studies and results of the analysis, it is obvious that the majority of the selected countries are yet to find a workable e-waste management strategy that will provide a sustainable solution to their e-waste concerns.

Results of the analysis show that the volumes of e-waste generated are fast exceeding the available infrastructure and recycling facilities in the countries examined, thereby driving e-waste streams to flow into illegal and informal recovery. On top of that, the absence of an integrated framework that could support the monitoring and management of toxic and hazardous wastes has also created additional problems in managing e-waste in the selected countries and calls for a generic e-waste policy approach.

In addition, the increasing demand for second-hand EEE, particularly in developing countries (China, Indonesia, India, and Malaysia) due to poverty and the continuing technological modernisation, has made these countries dumping grounds for e-waste from developed countries. For example, China’s Guiyu town is well-known for the informal recycling of printed circuit boards. Specifically, “metal-contaminated sediments and elevated levels of dissolved metals have been reported in rivers around the town of Guiyu” [85].

Furthermore, sophisticated facilities and infrastructure required for formal recycling of e-waste using efficient technologies are minimal or non-existent in the selected countries. Formal recycling is widely accepted as the best way to manage e-waste, which reduces greenhouse gas emissions and helps lessen the climate crisis. Thus, recycling e-waste will reduce air and water pollution associated with the illegal dumping of e-waste. By recycling discarded, unwanted, or obsolete EEE for new products, nations can further reduce the enormous health risks and environmental pollution associated with improper disposal of e-waste.

Therefore, to effectively manage e-waste in the selected countries, there is a need to develop generic structured policy approaches to tackle the e-waste problem in the selected countries and indeed across the world is required. These structured policies are projected to put in place formal systems and infrastructure for the recycling, management, and disposal of e-waste, taking into account country-specific issues.

One of the shortcomings of this study is that the information and analysis of previous studies are seen to be reality. This study is also limited to countries in the Asia Pacific region and considers the time limitation by the year of the articles found. Although the accuracy of some of the analyses in the present study is inescapably subjective, this study is a starting point for further research into various aspects of e-waste generation and management practices of the selected countries.

## 6. Recommendations

This study has exposed the current situation of e-waste generation and management practices of the selected countries. The following recommendations are suggested based on the findings of this study:

- E-waste regulations tailored to each country’s current situations should be enacted, recognising the lessons learned from more developed and experienced nations such as Japan, Switzerland, and South Korea;
- Extended producer responsibility (EPR) and 3Rs strategy should be implemented in EEE manufacturing regulations in all countries to support the production of simple, lightweight products, planned for reuse rather than obsolescence so that recycled materials can become resources for new products, thereby reducing the request for raw materials;
- Local government councils are key stakeholders in the management and recycling process and therefore incur major expenditures while handling e-waste. This, therefore, necessitates policymakers understanding of the determinants, drivers, and costs associated with e-waste collection and disposal;
- International integrated organisations should be established for checking specific e-waste material generation across the globe. This initiative will restrain the transboundary movement of e-waste across international borders.

### Policy Approaches

Although different countries have endorsed and passed their respective e-waste regulations in other to manage e-waste, implementing appropriate and structured policy approaches will support all efforts directed towards effectively managing e-waste across the globe. Firstly, it is critical to have stepwise, and well-thought-out policy approaches for effectively formulating and implementing e-waste regulations and guidelines. Such approaches have been found to be effective in more advanced countries such as Switzerland, South Korea, and Japan, as noted above. In view of the multidimensional socio-economic nature of emerging economies, it is vital to consistently assess and evaluate existing policies to identify gaps and areas for improvement. This technique has also been found to be effective in Australia. Secondly, when implementing e-waste policies, interdisciplinary research approaches need to be considered. This will allow policymakers to better understand and address the various health and environmental problems associated with e-waste management. Finally, we believe that the policy approaches of respective countries geared towards dealing with the persistent and challenging e-waste issues require a local and specific approach where inherent socio-cultural, economic, political, and environmental concerns of that country are taken into consideration.

## 7. Future Research

Future research should use a quantitative approach or other research methods and expand the number of selected countries to understand e-waste generation and management practices of countries in the Asia Pacific region. This will provide additional viewpoints in the management, recycling, and environmental management of e-waste in the regions.

## Figures and Tables

**Table 1:** E-waste generated around the world and per continent in 2016 [4].

Indicator	Africa	Americas	Asia	Europe	Oceania	World
Number of countries with sufficient data availability	47	32	44	39	12	174
Population (millions)	1064	931.8	4295	737.9	39.29	7068
WEEE total (Mt)	2.23	11.08	18.23	12.26	0.68	44.49
WEEE per capita, average of continent (kg/inh)	2.09	11.89	4.24	16.61	17.36	6.29
GDP total	2,309,676	24,061,119	26,870,635	21,347,978	1,552,169	76,141,597
GDP per capita, average of continent (USD/inh)	2170.5	25,819.7	6256.2	28,929.1	39,496.4	10,772.4

**Table 2:** The distinctive contents of e-waste.

Contents	Percentage in E-Waste
Metal	60%
Plastics	15%
Screens	12%
Metal-plastic mix	5%
Pollutants	3%
Circuit boards	2%
Cables	2%
Other	1%

**Table 3:** E-waste key statistics 2019.

Country	E-Waste Generated (kt)	E-Waste Generated (kg per Capita)	E-Waste Documented to Be Collected and Recycled (kt)	National Policy or Regulation in Place
Australia	554	21.7	58	Yes
China	10,129	7.2	1546	Yes
India	3230	2.4	30	Yes
Indonesia	1618	6.1	n/a	No
Malaysia	364	11.1	n/a	Yes

**Table 4:** Previous studies on e-waste conducted in the selected countries from 2005 to 2020.

Main Research Area	Countries				
	Australia	India	China	Malaysia	Indonesia
Electrical and electronic equipment modelling and e-waste estimation	√		√		
E-waste legislation and implementation practices	√	√	√		
Material flow analysis of e-waste	√				
E-waste generation estimation and recovery potential	√				
E-waste management practices	√	√	√	√	√
Extended producer responsibility (EPR) legislation	√		√		
E-waste recycling scheme	√	√	√		√
E-waste generation and mitigating measures		√	√	√	√
E-waste management systems			√		
E-waste social related issues		√	√		
E-waste environmental and health impacts	√	√	√		√

**Table 5:** Distribution of e-waste research in selected countries.

Main Area	E-Waste Categories Analysed	Study Location	References
Environmental management	General	China	[19]
Recycling	Computer, television, refrigerators, air conditioners, personal computers, mobile telephones, washing machines, home appliances and computers, printed circuit boards (PCBs), cathode ray tube, TVs and monitors, general	Australia, India, China, Indonesia	[55,68,71,88,92,93,94,95,96,97,98,99,100,101,102]
E-waste disposal and behaviour	Mobile phones, general	India, China, Malaysia	[90,98,103,104,105,106]
E-waste problems and challenges	General	Malaysia	[77]
Environmental and health impacts of e-waste	General	India, China	[85,97,107,108,109]
E-waste legislation	Household hazardous waste, general, computers, printers, mobile phones, home appliances	Australia, India, China, Malaysia	[13,44,67,93,94,95,110,111,112,113,114,115,116,117,118,119,120,121,122,123,124,125,126]
E-waste management	Printed circuit boards (PCBs), televisions, computers, printers, and IT peripherals, television and computer waste, photovoltaic panels and battery energy storage systems, mobile phones, home appliances	Australia, India, China, Malaysia, Indonesia	[18,21,22,28,49,70,79,80,83,86,87,95,96,113,114,115,116,117,118,119,120,121,122,123,124,125,126,127,128,129,130,131]
E-waste generation	TV sets, refrigerators, washing machines, air conditioners, microwaves, vacuum cleaners, dryers, personal computers, heaters, mobile phones	Australia, India, China, Indonesia	[9,28,46,56,65,72,83,131,132,133,134,135,136,137,138]

**Table 6:** Findings from the analysis of the contents.

Country	Findings
Australia	- Lack of clarity on the roles of key stakeholders involved and the recycling and material recovery targets. - Minimal research has been undertaken to assess the effectiveness of e-waste policy management strategies.
China	- Various issues geared towards developing a sustainable recycling system still need to be addressed.
India	- Recycling of e-waste is heavily dominated by the informal sector, and only a few approved e-waste recycling facilities are available. - Formal process of e-waste recycling and treatment is still rather slow.
Indonesia	- Infrastructure and workable systems to quantify, recycle, monitor, and handle e-waste are lacking. - The informal sector illegally collects, treats, and disposes of discarded EEE, causing huge environmental and health issues.
Malaysia	- Several material recovery facilities have been built, but it still faces significant issues in managing the ever-increasing amount of e-waste generated.

## Data Availability

Not applicable.
